# Pathway of Trends and Technologies in Fall Detection: A Systematic Review

**DOI:** 10.3390/healthcare10010172

**Published:** 2022-01-17

**Authors:** Rohit Tanwar, Neha Nandal, Mazdak Zamani, Azizah Abdul Manaf

**Affiliations:** 1 School of Computer Science, University of Petroleum & Energy Studies, Dehradun 248007, India; 2 Department of Computer Science and Engineering, Gokaraju Rangaraju Institute of Engineering and Technology, Hyderabad 500090, India; neha28nandal@gmail.com; 3 Department of Computer Science, New York University, New York, NY 10012, USA; 4 Independent Researcher, Kuala Lumpur 54100, Malaysia; azizahmanaf18@gmail.com

**Keywords:** fall detection, fall prediction, fall prevention, fall risk factors, gait assessment

## Abstract

Falling is one of the most serious health risk problems throughout the world for elderly people. Considerable expenses are allocated for the treatment of after-fall injuries and emergency services after a fall. Fall risks and their effects would be substantially reduced if a fall is predicted or detected accurately on time and prevented by providing timely help. Various methods have been proposed to prevent or predict falls in elderly people. This paper systematically reviews all the publications, projects, and patents around the world in the field of fall prediction, fall detection, and fall prevention. The related works are categorized based on the methodology which they used, their types, and their achievements.

## 1. Introduction

According to the World Health Organization [1], approximately 28–35% of people with an age of 65 fall every year. The count further increases to 32–42% for people of age 70. With the rapid rise in the number of elderly people, the demand for supportive healthcare systems has also increased. The advancement in the fields of sensors, cameras, and communication makes it feasible to develop more efficient and optimized healthcare systems. Moreover, financial support from the respective governments motivates researchers to help elderly people through their valuable research [2]. Research in the medical field shows that a human being’s process of aging leads towards a decreased walkability in elderly persons along with bringing down the physiological and nervous system function. Therefore, the probability of being injured during a walk becomes greater, which can cause several anile diseases. The prediction and evaluation of fall risks are very important given the surging number of aged people [3]. The impact of falls in the elderly is extensive and occurs across the world [4,5]. The process of fall prevention includes knowing and assessing the parameters responsible for a fall, predicting the possibility of a fall, and then not letting the fall happen. The process may include medical and paramedical treatment to fine-tune the fall parameters, the use of some aids, and some similar methods. It is very difficult to prevent a fall; however, long-term treatment may help in achieving fall prevention. Fall intervention is a set of techniques that help prevent future falls. Techniques that include exercise, home modification, and medication are carried out under clinical or self-administration with the aim of fall prevention in elderly persons [6].

### 1.1. Fall Risk Factors

**Figure 1:**
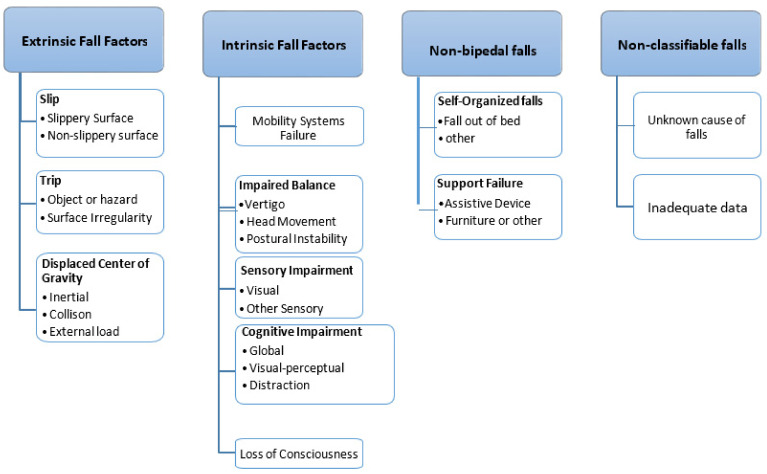
Fall risk factors [11].

Understanding the possible risk factors responsible for falls in elderly persons is required. A better understanding of these risk factors will help in developing a better fall prevention system. Numerous factors related to biology, behavior, demographics, and environment are there that can be a cause of falls for an elderly person [7]. A list of risk factors has been identified through the study of relevant and published literature, as shown in Figure 1. Numerous causes are responsible for the fall of an elderly person or patient. Physiological conditions and falls from the bed are the most common cause of the fall [8,9,10]. The authors in [11] designed a reliable and flexible method for the classification of falls in the elderly. Along with that, the operational definitions for types of falls were also provided. In the proposed three-level hierarchical classification scheme, the first level consists of four major classifications. Each major classification has further subcategories which are further divided into other subcategories of level three. The detailed categorization is shown in Figure 1.

### 1.2. Types of Fall

**Figure 2:**
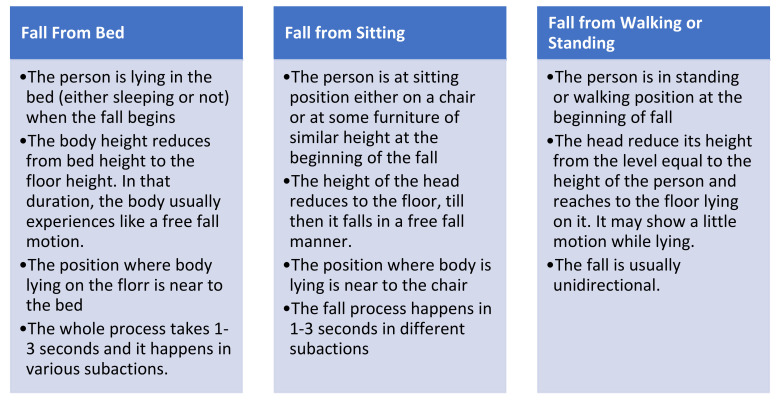
Types of falls [8,9,10].

Categorizing falls used to be a great issue until the 1990s. A lack of consensus among researchers was the biggest hurdle. Most of the categorization then was based upon the factors responsible for falls. Depending on the position before a fall, a fall was considered to be of three general types, as described in Figure 2.

### 1.3. Fall Detection/Prevention Approaches

A list of technologies has been developed by researchers to detect and prevent the occurrence of falls in elderly people. Numerous techniques have been used to handle the problem of falls among elderly people. These approaches are based on the integration of machine learning, IoT (Internet of things) devices, imaging techniques [12], etc. The continuous monitoring of the elderly person using either wearable or non-wearable devices and finding the probability of their fall in advance is known as fall prediction [13]; however, fall prediction is more concerned with the detection of fall risk factors. It requires a highly accurate prediction mechanism that could respond instantly in no time. However, it is not easy to achieve, but an accurate prediction will significantly contribute to preventing elderly persons from the after effects of falls. Fall detection is the process of finding out that an elderly person has experienced a fall and then sending some alarm signal to let medical professionals know about the incident. Various incidents might give an illusion of a fall, such as sitting on a chair from a standing position, bending on knees to pick something up, etc. The process is expected to differentiate actual falls from false falls and then send an alarm to pre-specified people or locations instantly. The intention is to send help to the elderly people after the fall as soon as possible so that after effects can be minimized.

**Figure 3:**
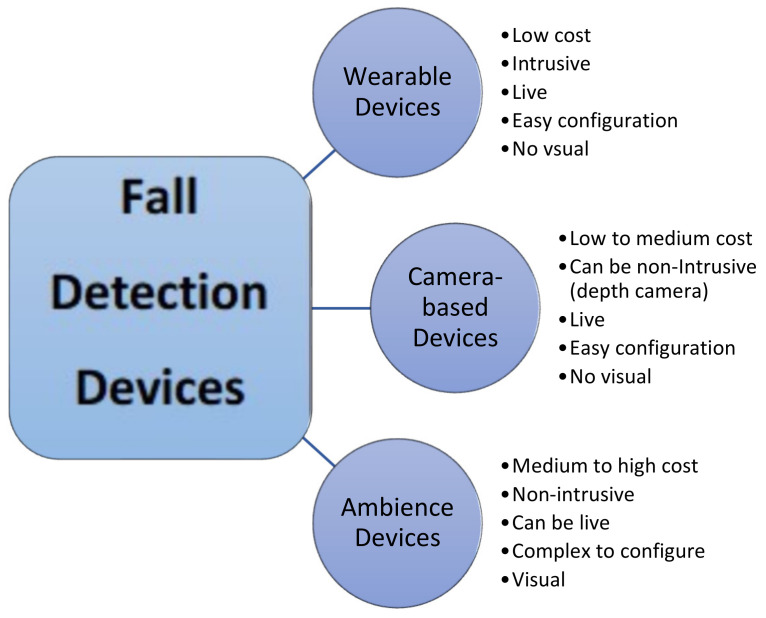
Fall detection approaches [2].

**Fall detection:** fall detection techniques can be classified into three basic categories: (i) wearable devices, (ii) camera-based devices, and (iii) ambience devices. The categorization of fall detection is presented in Figure 3. In the wearable devices approach, some wearable gadgets or garments need to be worn by the people at risk of a fall. These devices sense the information regarding the body posture or the movement and then some algorithm processing this information decides whether it is a fall or not. The decision is then communicated to the pre-specified caregivers. However, the use of wearable devices seems to be very intrusive and an extra overhead to some users. They do not want to bother to keep on wearing any device all the time. Moreover, there is an issue regarding the placement of the device. Some activities, such as sleeping and walking, might displace the device from its original location and may result in less accurate results.

The camera-based approach seems to overcome some of these issues. The cameras are placed at selected locations so that continuous monitoring of the elderly people can be performed passively. Unlike sensors, it is possible to assess and analyze more than one feature using the camera. These types of systems were less preferable initially when the cost of the camera used to be very high. Additionally, the data captured through these devices can be stored for later analysis and reference. In the ambience device approach, a series of sensors are installed in the vicinity of the related persons, such as a wall, floor, bed, etc. The data are gathered from these sensors and, using that input, an algorithm decides whether there is a fall or not. Consequently, the incident is reported to the caregivers. Since there is no need to wear any sensor, the related person is not concerned about any type of overhead.

A variety of devices from different manufacturers are available in the market that send alerts when a fall occurs. According to a survey, the number of automatic systems for detecting falls will cover 60% of the fall detection systems market by 2019–20. The compound annual growth rate (CAGR) is expected to be approx. 4% from 2019 to 2029 [14]. Governments are investing more in research related to fall detection devices so that the major portion of their budget that is used in medical care and treatment of after-fall injuries could be minimized. These devices differ in their location of the mount, response time, size, etc. Some of the devices are listed [10,11,12,13] below:

1. MobileHelp
2. Medical Guardian
3. LifeFone
4. Bay Alarm Medical
5. GreatCall Lively Mobile Plus
6. Apple Watch

**Fall prevention:** preventing falls in elderly people is something that cannot be guaranteed and achieved 100%. It can be used as an activity for ensuring that the targeted person is in a minimal risk zone. It is performed through continuous monitoring and periodically assessing the status of identified fall risk factors. If the observed values for those parameters lie in the acceptable range, then the targeted people might be assumed to be in the safe zone. The list of activities [15,16] that can be performed for fall prevention can be listed as:

- Notice if they are holding onto walls, or something else, when walking, or if they appear to have difficulty when walking or arising from a chair.
- Talk about their medication.
- Complete a walk-through safety assessment of their home.
- Enlist their support in taking simple steps to stay safe.
- Discuss their current health conditions.
- Perform regular checkups of the eyes and spectacles.

## 2. Methodology

**Figure 4:**
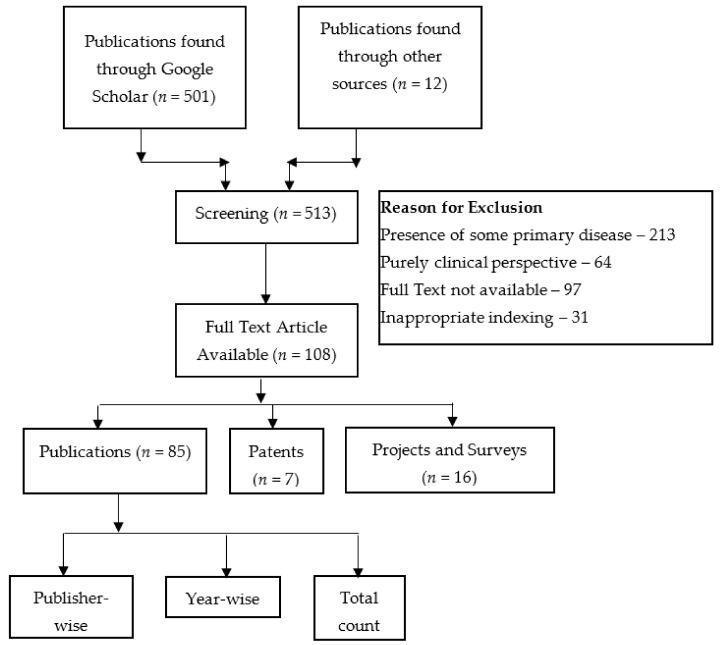
Review methodology.

This section discusses the methodology followed for carrying out this work. The literature studied comprises the work completed as publications, patents, and funded projects or surveys in this domain in the specified time duration, as shown in Figure 4. The query used for searching is a Boolean “OR” combination of the terms “Fall Detection”, “Fall Prediction”, and “Fall Prevention”, and it should appear in the title of the publication. A number of projects/surveys and patents completed in a window of two years starting from the year 1991–92 was sought out. The process was repeated for the subsequent two-year periods until 2020. Similarly, the number of publications was identified using Google Scholar. Additionally, the publications were also categorized according to the different publishers, including Springer, Elsevier, IEEE, etc. The publications were further grouped based on the technology used to detect/predict/prevent falls. The articles that were purely concerned with clinical research were excluded. Additionally, the articles where falls were a secondary concern, and the primary concern was some pre-existing disease, were not included. The non-availability of full text and indexing in some inappropriate databases were also considered as part of the criteria for exclusion.

## 3. Research Publications

To make the review process more systematic, the research publications that have been studied are placed in various categories depending upon the underlying technology they are focused upon. Before discussing the research publications in various categories, it is better to describe the parameters used for the evaluation of an algorithm used for fall detection or prevention.

### 3.1. Evaluation Parameters

A fall detection or prediction model needs to be tested for its effectiveness at analysis. The following four parameters [15,16] are used to evaluate a given model:

(i) Sensitivity: the system can detect falls correctly. It is defined as the ratio of the number of falls correctly classified and the total number of falls as follows:

(1) $$\mathrm{Sensitivity}=\frac{\mathrm{TP}}{\mathrm{TP}\;+\;\mathrm{FN}}$$

where, TP = Falls correctly identified, and FN = Fall not detected by the model.

(ii) Specificity: the system can avoid false alarms (detecting an event similar to a fall, which is not a fall in actuality). It is calculated using ADL (activities of daily living) as follows:

(2) $$\mathrm{Sensitivity}=\frac{\mathrm{TN}}{\mathrm{TN}\;+\;\mathrm{FP}}$$

where, TN = Number of ADL coorectly classified, FP = Number of False Falls

(iii) Accuracy: accuracy is the capability of a model to correctly identify actual falls and to recognize falls false as well. It is calculated through a balanced calculation of sensitivity and specificity:

(3) $$\mathrm{Accuracy}=\frac{\mathrm{Specificity}\;+\;\mathrm{Sensitivity}}{2}$$

(iv) False positive rate: this is the number of false falls identified as actual falls per hour. It is calculated as a ratio of the number of false falls to the total time of recording:

(4) $$\mathrm{False}\;\mathrm{Positive}\;\mathrm{Rate}=\frac{\mathrm{FP}}{ADL\;time\;\left(in\;hrs\right)}$$

### 3.2. Cell Phone-Based Approaches

A simple system for fall risk prediction is developed in [3] using a cell phone along with a three-dimensional accelerometer. Practically, it is less expensive to use the accelerometer to monitor a human walking as an object. Along with the proposed work, the authors defined gait symmetry and stability under the data conditions of acceleration. The proposed gait assessment model was capable of analyzing and evaluating the stability and symmetry of an individual’s gait. The proposed gait assessment model could predict the fall risk of a walking object correctly. The improved results for the performance and efficiency were obtained, justifying the effectiveness of the work. The problem of fall prediction is a manifold one, whose solution demands balanced coordination of behavioral, physiological, and environmental parameters.

Fortina and Gravina [12] designed a system comprising a smartphone and wearable accelerometer that sends an alarm when a fall is detected in real time. The system was capable of triggering fall incidents using different alerting modalities, providing emergency services with a notification in no time. The approach was tested on 20 subjects and the results reported an 83% specificity, 97% sensitivity, and 90% precision. The fall detection system in the future would be improved in terms of design and evaluation and become better because of this work. Research on the invention of modest wearable devices for blood pressure checking to detect orthostatic hypotension and the associated fall risk is almost nullified, however, although the research on using smartphones as devices to detect falls is in transit, and certain limitations are still challenges that need to be resolved, as listed below:

- It is doubtful whether the quality of the built-in sensors of cell phones [17] is good enough to properly identify falls. The accelerometer sensor of smartphones have dynamic ranges of up to ±2 g, but the level required for a fall detection device to produce an appropriate result is ±4 g to ±6 g (1 g = 9.8 m/s^2^).
- The limited battery life (only a few hours) of smartphones on heavy usage is a major concern [17]. Past studies show that battery consumption rises to more than double when three sensors are used simultaneously. Using power-saver mode appears to be a genuine solution, but the performance would be affected considerably.
- Smartphones are not designed and developed purposefully for detecting falls [1]. The various compatibility and operational issues result in a compromise with accuracy when used in real time.
- The positioning of mobility sensors significantly impacts the behavior of fall detectors. The accuracy of the smartphone-based fall detection systems demands its mounting or placement at some particular and unnatural position, usually the chest or wrist [15]. However, this mandate of positioning either produces discomfort to the user or compromise with the accuracy achieved. Moreover, an additional device is needed to carry and position the smartphone at the desired point. It makes the product less attractive overall.

### 3.3. Sensor-Based Approaches

**Figure 5:**
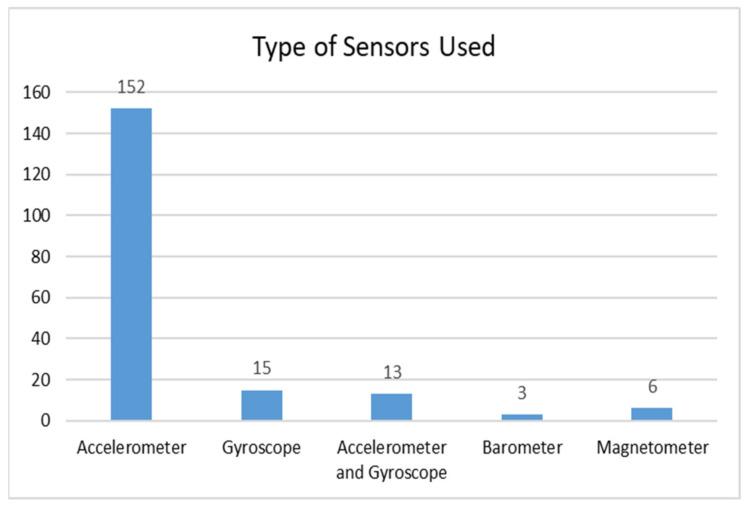
Types of sensors used in fall detection.

The use of accelerometer and gyroscope sensors either alone or in pairs has been the preferred choice of researchers to detect falls. In some research, the existing sensors of the devices are being exploited for fall detection, while in others, the desired sensor(s) is/are connected externally. Figure 5 shows the use of the different types of sensors in fall detection and prediction. The accelerometer sensor was used in 86% of the research works related to fall detection or prediction. Only 5% of the researchers used a barometer and magnetometer for fall detection.

The problem of falls in the elderly is renowned and hazardous throughout the world. A delay in fall assistance may result in practical damage to the elderly person along with a decrease in movement and ease of living. The authors of [18] suggested a novel system to detect falls in aged people using the IoT. Their approach was based on utilizing energy-efficient wireless sensor networks, cloud computing, and smart devices. The wearable device was designed by embedding a 3D-axis accelerometer into a 6LoWPAN (low-power wireless personal area networks) device. The real-time data were collected from the movement of elderly people. To detect falls with improved efficiency, a decision tree-based big data model, along with a smart IoT gateway, is used for processing and analyzing sensor data. The moment a fall is detected, the system reacts by sending an alert message to the caregivers or emergency services chosen for providing care. The data are managed and stored in the cloud. The medical professionals can use that data for further analysis. Additionally, there is a system service that generates another machine learning model based on these data to adapt to future falls. The experimental consequences were improved fall detection success rates, measured using accuracy, gain, and precision.

Gait analysis and the monitoring of mobility are usually performed using accelerometers and gyroscopes in wearable systems. Most of the researchers recently have worked on obtaining and analyzing the data from accelerometers and gyroscope sensors for the assessment of fall risks [17,19]. Bourke et al. [20] analyzed different permutations of the magnitude of acceleration, sensor velocity, and body posture and, based on that, a fall detection system was developed. They observed that the maximum value of fall sensitivity along with the lowest value of the false positive rate was achieved when the three parameters were fused and used with a triaxial accelerometer. Bianchi et al. [21] developed a wearable device by utilizing an accelerometer along with a pressure sensor that mounts on the waist. Different variants of fall scenarios occurring indoors as well as outdoors were tested to minimize and avoid false alarms. The results revealed that false positives occurring under general circumstances are reduced considerably with the usage of the barometric sensor. As with other usual research, the authors simulated the testing environment, and healthy young people were used for testing the device. Ease of wearing is a prime characteristic of fall detection with wearable devices because of their continuous use for a long time. A study on the wearable devices found that, in a trial with a case that involved an enclosed waist-mounted device for fall detection performed on aging adults for three months, the device was transferred to different body locations because of discomfort and bruising [22]. Thus, along with small size, comfort is also a main factor that should be focused upon. The devices should not cause discomfort even if they are used for a long time and attached to the same location. Howcroft et al. [23] analyzed the performance of using two wearable sensors together in predicting fall risks. Two sensors, i.e., pressure-sensing insoles and accelerometers, four locations of accelerometer, i.e., head, left, pelvis, and right shank, and choices of three models, i.e., support vector machine (SVM), naïve Bayesian, and neural network. The observations reported that the best input can be provided for predicting falls when gait assessment is performed using multiple sensors, such as with a hybrid of the posterior pelvis, neural network, and head and left shank accelerometers. Some researchers [24] have invented a novel approach to avert the fall of a user by governing a passive intelligent walker as per the walking attribute of the user. These sensors are connected with an aid device for walking to identify gesture movements and the sensor’s distance from a person. These types of sensors usually have a short range and a high rate of false alarms, with an individual stepping away from the walker being misunderstood as a fall. Another researcher [25] worked on the prevention of bedside falls and introduced a “Bed-exit” alarm. The proposed system utilizes pressure sensors. The pressure sensors are embedded on the side rails of the user’s bed to sense the movement of an individual if they move out of the bed. A threshold value is to be set for the pressure sensor which, if exceeded, leads to an alarm going off to prevent the fall. For interactions with fall prevention exercise games, the available ambient sensors are often utilized. Researchers, Pisan et al. [26] and Kayama et al. [27], proposed systems that utilize Microsoft Kinect sensors with a game invented for older adults. The proposed game helps to identify the functional and cognitive changes in the patients by carrying out different physical and cognitive tasks. Multi-tasking has been embedded because it is proven to be a reliable predictive factor for future falls. Tong et al. [28] presented an HMM (hidden Markov model) method utilizing a triaxial accelerometer for fall prediction. Additionally, the proposed work again has not been tested and analyzed on real-life scenarios and elderly people who can be an example of people who are fall prone.

The solution to wrist-worn fall detection, and its development and assessment, has been presented in this paper [29]. Several different types of signals and direction components were collaboratively utilized along with machine learning methods to find out the best approach for fall detection. The sensors included a gyroscope, magnetometer, and accelerometer, the directions utilized were vertical and non-vertical, and the signals included velocity, displacement, and acceleration. Data for the work were collected from 22 volunteers for both fall and non-fall movements. With machine learning methods, an accuracy of 99.0% was achieved along with 100% sensitivity and 97.9% specificity. Additionally, the work has been tested with threshold methods, and a 91.1% accuracy was achieved along with a 95.8% sensitivity and 86.5% specificity. In the view of practical applications, the benefits of machine learning methods have been elaborated upon by the prolonged tests of a volunteer wearing a fall detector. Work has been proposed in [16] to detect falls in aged people in indoor environments. This was an IoT-based system that takes advantage of low-power wireless sensor networks, cloud computing, and big data. For its implementation, a 6LoWPAN device wearable was used in which a 3D-axis accelerometer had been embedded, which can collect data from aged people’s movements. The reading collected by the sensor was analyzed utilizing a decision tree-based model. An alert is activated if a fall is detected, and the system reacts automatically by providing notifications. Lastly, the services will be provided built on the cloud. The system provides a service that leverages these data for building up machine learning models every time a fall is detected. The work showed very effective success at achieving results within the parameters of precision and accuracy. The work presented in the survey [30] utilized a depth sensor. A unique process to identify levels of fall risk has been implemented. This procedure of level identification is an enhancement of fall detection. The proposed algorithm showed effective performance results. The different and many suggestions along with solutions are present in the form of several tools, resources, and assessments for intervention, but falling is one of the major health problems which can occur to an individual. In today’s time, it is considered highly desirable to go for health care if a severe fall happens.

The proposed model [31] is a working sub-model for the real-time monitoring of heart attacks and falls of a patient. To develop this system, an Arduino UNO and Arduino NANO-based process has been included as the architecture, with pulse and accelerometer sensors. The key concept is to gather the data related to health from time to time, and the data collected are to be made available utilizing a real-time interface called Thingspeak. Within this process, the person can be invigilated from time to time without any disturbance. The proposed model is also utilized to deliver notifications at the time of emergency with GSM (global system for mobile communication) technology, which is combined with the Arduino architecture. This model will be greatly helpful for elderly people, Frankenstein syndrome patients, or patients with a history of heart attacks because of genetic disorders. Other work [32] shows a health monitoring solution that identifies the occurrence of accidental falls in the elderly. The technique of fall detection implements sound- and accelerometer-based detections for valid fall occurrence. Fall detection based on an accelerometer is instrumental for the valid detection of fall occurrence. However, it has been shown that an accelerometer individually is not enough for fall detection because an accelerometer is affected by misinterpretations of routing motion activities, categorizing them as falls. To detect the pressure of sound from a resultant fall, the utilization of sound sensors has been integrated, but the pressure of sound is not enough to be utilized as a trustworthy fall indicator. Therefore, a method for the detection of falls based on fuzzy logic has been presented to activate the sound sensor and accelerometer’s output signals, and the utilization of a sound pressure detector to verify the signal provided by an accelerometer can lower the incorrect fall detection rate of every day falls from 1.37 to 0.06. Choosing a particular paradigm, given the many approaches for detecting falls and ADL, needs some parameter to ease the selection. Power consumption is one such parameter, especially when dealing with embedded systems with limited constraints. Most of the wearable as well as non-wearable devices involve classification as one of its essential steps. Generally, machine learning algorithms or threshold-based approaches are exploited for classification purposes. The low computation needs combined with the moderate classification performance of threshold-based approaches creates a trade-off with the machine learning algorithms that normally demand high computation and offer better classification performance. A solution was presented for this problem in [33] that matches the power constraints of embedded systems. The method exploited advanced signal processing to find the maximum correlation of the unknown event within the available set of fall and ADL signatures. The power requirements were reduced by adopting a modified alignment strategy along with a normalization procedure specifically targeting the computational requirements. The method was able to satisfactorily classify an unknown event belonging to a specific class of events. Paper [34] discusses UWB (ultra-wide band) sensors, which are both environmentally and practically based on radar and are non-wearable, as a solution. Specifically, we are concerned about the impact of unsupervised changes in detection techniques on UWB sensor information to detect falls. Furthermore, accelerometer sensor information is also used for assessing the oversimplification of our unsupervised method for fall detection. Planned techniques are assessed using UWB sensor information sets obtained from an Australian E-Heath research center (i.e., Living Lab) and publicly accessible accelerometer sensor information sets. Results produced capable outcomes. Work [35] shows a stance recognition-based fall discovery framework for wellbeing observations, predicated based on keen sensors worn from the body function using personal networks. If it can be determined that this has the best range limit, when incidental falls occur, it could be successfully utilized in combination with an android gadget. By aggregating the full-time information and learning of an accelerometer, cardio tachometer, and other intelligent sensors, a fall might be calculated and separated from our ordinary lifestyle. The technique concerning the planned framework has been clarified in a much more feature in the paper. The planned framework accomplishes a 99% exactness rating by utilizing exclusive sensors similar to a temperature sensor, a circulatory strain level-checking sensor, and a cardio tachometer.

The work completed in paper [36] shows how one of the projected solutions in the literature has been modified for use with a smartwatch on a wrist, solving some problems, and updating part of the procedure. The testing includes a publicly accessible dataset. The results point to numerous enhancements that can be adapted for the target population. Other work [37] is focused on designing and developing a live system capable of detecting falls in humans. When a fall occurs, it would be able to alarm the concerned person so that the after-fall damages can be minimized. This can be used to reduce the damages at construction sites and in industry as well. The setup was assembled as a low-cost gadget using a MEMS (microelectromechanical systems) motion sensor (MPU-6050) and a GSM or RF (radio frequency) to send data. The mounting location of the gadget is chosen in such a way that a minor change in the center of gravity of the subject can be noticed.

The information is then processed and analyzed to detect the occurrence of falls. In this paper [38], an approach is presented that detects the fall of an elderly person while moving inside the house or indoor premise and provides their exact location. A sensor-based fall detection method is used to detect the occurrence of falls and the location is provided using an artificial neural network. The work conducted in [39] was based on the Internet of things (IoT), and focused on the development of an energy-efficient wearable sensor node. A lightweight, energy-efficient, small-size and flexible device was designed for detecting falls. The design was a consequence of an exhaustive study on the parameters that affect energy consumption in IoT devices (wearable devices). The scope of research on ambient assisted living using smartphones motivated the researchers to work in this area. It was concluded from various approaches that wearable devices perform better at identifying falls from ADLs. These systems are tested in a controlled environment and optimization is performed for a given set of sensor types, sensor positions, and subjects. A self-adaptive pervasive fall detection method is proposed in this work. The work proposed is robust to the heterogeneity of practical situations in life [40]. The authors in [39] proposed an RNN (recurrent neural network)-based human fall detection method. The ability of the network to work with acceleration measurements from sensors means that it has the appropriate tools for the task. Study [41] presented an IoT fall system for the fall detection of elderly people that uses the benefits of IoT. The proposed system shows a 3D-axis accelerometer added into a 6LoWPAN wearable device with the capacity of measuring the movements of elderly volunteers as data. Table 1 shows the specificity (SP) and sensitivity (SE) achieved by various researchers. The research work considered in this table has exploited the accelerometer sensor for detecting falls. It can be observed from the table that various researchers have succeeded at achieving 100% specificity and sensitivity by using an accelerometer to detect falls [42].

### 3.4. Camera-Based Approaches

In fall detection and prediction systems, there is a high usage of camera-based sensors [53,54]. For monitoring the routing activities of any individual, distinct cameras are used in such systems. Along with the pros, these systems also have some cons, such as budget and privacy, and they are unable to track beyond the camera range. Another fine example of ambient sensors is proximity sensors, which are utilized for fall detection. Bian et al. [55] utilized a single-depth camera to introduce a novel approach for fall detection in which key joints of the person’s body are to be analyzed. This newly developed approach utilized an infrared-based depth camera which can work in dark environments. However, the invented approach is not able to identify the falls that end with the person lying on the furniture. Paper [54] planned an integrative replica of fall motion recognition and fall severity level assessment. The detection of fall motion and the presentation of data in a continuous stream, with the time-sequential frames fifteen body joint positions, have been obtained from Kinect’s 3D camera. Some features are extracted and fed into a designated machine learning model replica. Compared to existing models, which rely on inputs of the image depth, the planned method resolves the background uncertainty of the human body. The experimental outcome confirmed that the planned method of fall detection achieved 99.97% accuracy with zero false negatives and was robust compared to the state-of-the-art approach because it utilized image depth.

The work completed in [56] suggested a method for detecting falls using the 3D skeleton data received from a Microsoft Kinect. The technique utilized the accelerated velocity of the center of mass (COM) of different body components and the skeleton data as main biomechanical features and applied long short-term memory networks (LSTM) for detecting a fall. Unlike other similar methods, it does not require the mounting of a sensor on any body part of the elderly, people preserving their privacy. The method was tested and validated on the existing dataset and was found to be effective in fall detection. Since no special mounting of sensors is required, the device can be used for detecting falls in elderly people at home. This paper [57] discusses an intelligent fall detection system based on video. The first step is to extract the silhouette of a person using the background subtraction method; a collection of features is then evaluated to estimate a fall. The head position is estimated using a new technique and its virtual velocity is computed using an FSM (finite state machine).

For the expansion of systems that are human interactive, the visual human action classification is important. The work [58] enquires about a human stage classification that is image based, with a walking support system to increase safety. The paper [59] presented a real-time system that is very fast and more accurate and able to identify falls in videos taken by cameras. A new spatial and temporal variant-based aspect is presented which comprises the geometric orientation, the location of a person, and their discriminatory motion. The datasets used for the study are different cameras that fall with two and three classes. An accuracy level in the range of 99.0 to 99.2 has been achieved. A comparison of nine methods has been conducted and the effectiveness and improvement of the presented approach with the dataset have been given in the work.

### 3.5. Survey/Questionnaire

The authors in their work [2] have reviewed the existing fall prediction methods and strategies for old people and patients. Based on the approaches using sensors, the techniques for detecting falls are categorized into three domains namely, “Wearable Devices”, “Ambience Devices”, and “Camera-Based”. Each class is subdivided further based on their fundamental principle of working. The advantages and disadvantages of each category have been listed along with the remarks for further improvements. Similarly, in [8], the authors have conducted a systematic survey of existing systems for predicting falls in the elderly. The shortcomings and the challenges listed by the authors help to design effective implementation techniques for fall prevention and prediction. One of the recent surveys highlighted a crucial point regarding wearable devices, namely that 32% of the users usually stop wearing them after 6 months and almost 50% stopped their usage completely after a year [60]. Therefore, a requirement of research must be to scrutinize the functionalities of wearable devices, such as modishness, budget, reliability, and flexibility, to increase its demand among customers. Questionnaires and assessments are often a part of clinical fall risk analysis that can analyze posture, cognition, and other important fall risk factors [61]. Questionnaire and assessment analysis provides a sample and snapshot for analyzed fall risks. They are usually subjective and utilize threshold assessment scores to categorize an individual as fallers and non-fallers [62,63]. However, fall risk flow should be modeled based on a continuum, and include categories of risk, such as low, moderate, or high fall risk. Modest sensors and distinct health tools can be utilized to perform the longitudinal monitoring of aging adults who can provide an effectively accurate assessment of fall risk. Shany et al. [64] introduced the utilization of wearable devices, such as sensors, for fall risk, especially under supervised and unsupervised environments. However, discussions about the testing, validation, and maintenance of different methodologies and real-life fall implementations are not being discussed in this work.

Another work in [65] shows a methodic review according to PRISMA (preferred reporting items for systematic reviews and meta-analysis statement) principles. Twenty-two studies out of eight hundred and fifty-five were studied for this work. The features which were extracted from the study were the outcome variables, fall prediction models, sensing techniques, and assessment activities. Four major sensing technologies, i.e., cameras, pressure sensing, laser sensing, and inertial sensors, were found to be useful for predicting fall risk accurately in elderly adults. The work presented accuracy levels in the range of 47.9% to 100% because of modeling techniques and kinematic parameter variations. Several sensor technologies have been used in fall risk analysis in elderly adults. It can be said that the devices are very valuable for providing an easy-to-handle and accurate analysis. In the future, it is necessary to find out ways to diagnose fall risk by using sensor technology. One of the major concerns of healthcare in several communities, specifically with elderly people, is unintentional falls. Related surveys have found that sensors, cameras, and sensor-based approaches are used to develop systems that can classify fall detection with human beings. The work presented in [66] elaborates upon three parameters, i.e., prevention, assessment, and intervention, which are shown as a three-tier model. This work has been conducted to bring together innovative tools, proactive programs, and technology that have been constructed for fall prevention. The realization of the resources will intensify the clinician’s capability to precisely assess gait and balance, with the help of which the risk of falls can decrease. Research work [67] concentrates on falls in the elderly and how elderly people can be helped with fall prevention. As per the survey, 20% of all the elderly who have fallen remained on the ground for more than an hour. Moreover, 50% of the elderly people who suffered from falls die within 6 months of it, even if there are no physical injuries. The psychological effects can also lead to death. More than 50% of elderly people suffer a fall far from home where installed fall detection systems cannot reach. One of the top reasons for fatal as well as non-fatal injuries in elderly people is due to falls.

Fall frequency within one year calculated using time-to-time monitoring has defined the status of falls for 7/153 fallers or non-fallers. Based on [68] and their analysis of 718,582 turns, prospective fallers turned less frequently, took a longer time to turn, and were not very reliable in terms of their turn angle (*p* = 0.007, 0.025, and 0.038, respectively). Prospective fallers walk slower, use up less time walking and turning, and have extra time occupied in sedentary behavior (*p* = 0.043, 0.012, and 0.015, respectively). Those who have less control over their gait and turning abilities might attempt to decrease the risk of falling by restraining exposure and implementing advisory progress strategies while turning. As there were hardly any differences in general active rates among fallers and non-fallers, turning ability and gait may lead to an elevated risk of fall. Falls of patients and other injuries related to falls remain a concern of safety. The JHFRAT (Johns Hopkins fall risk assessment) device [69] has been utilized to perform untimely risk detection, which is meant to anticipate physiological cascades in adult patients. Psychometric properties in keen care settings have not been so far completely recognized; this revision sought to fill that space. The presented results showed that JHFRAT is reliable, with negative predictive validity and high sensitivity. Positive predictive validity and specificity were lower compared to the expectation.

An assessment for the identification of fall risk [70] is usually performed in hospitals and environments, such as the laboratory. Instead of these assessment testing methods, a passive monitoring solution in the home would be a cheaper and less time-consuming option. As sensors become more readily accessible, a machine learning replica can be utilized for the huge amount of information they create. This is useful for the finding, prediction, and risk determination of falls. In this review, the increased complexity level of sensor information required analysis, and the machine learning methods used to decide the risk of falling were analyzed. The latest research on utilizing passive monitoring in house has been discussed, whereas the viability of active monitoring by utilizing wearable and vision-based sensors has been measured. The comparison of methods, such as prediction, detection of falls, and mitigation of risk, has been conducted. This study [71] proposes a technique to analyze the ways in which elderly adults at high falling risk interact with the smart rollator, i-Walker, to navigate indoor, flat environments. The smart rollator is a sensor and actuator prepared and able to collect data for several hours. In [72], a multi-parametric score based on consistent fall risk assessment tests, along with medication, the history of a patient, their motor skills, quality of sleep, and environmental factors was planned. The resulting entire fall risk score reflects entity changes in vitality and behavior, which are triggers for fall prevention interventions. The deployment and evaluation of the system has been conducted in a pilot learning program for 30 elderly patients over 4 weeks. Another paper, Ref. [73], depicts a person in motion as a scatterer using time-variant (TV) speed, TV vertical motion angles, and TV horizontal motion angles of scatterers in motion. In addition, we obtained TV angular parameters of every moving scatterer, such as the departure angle of elevation, the azimuth departure angle, the arrival angle of elevation, and the azimuth arrival angle. Moreover, TV unit vectors of the departure of transmitted wave planes and unit vectors of the arrival of the received wave planes are obtained. Additionally, showing the Doppler power spectrum uniqueness of such channels provides a closed-form explanation of the spectrogram of complex channel growth. The precision of the analysis is determined using simulations. The paper contributes an initiative for implementing to device-free monitoring of indoor activity and systems of fall detection.

Study [74] collects and analyzes technological solutions that exist for the assessment of fall risk with several sensor-based technologies. This work also presents an easy solution for fall risk assessment and provides a design based on the concept for the integration of solutions based on the sensor for the Finnish National Kanta Personal Health Record. Paper [75] shows that older adult falls result in substantial medical costs. The calculation of medical costs attributable to falls provides important data about the problem’s magnitude and the potential financial outcomes of effective prevention strategies. The objective of the study [76] was to expand a fall risk mobile health (mHealth) app and to decide the applicability of a fall risk app in healthy and older adults. A fall risk app was created which carries a health history questionnaire and five progressively challenging mobility responsibilities to determine individual fall risk. An iterative design–evaluation process for semi-structured interviews was created for resolving the usability of the app on a smartphone and tablet. Participants also completed a systematic usability scale (SUS) assessment. Standing-level falls [77] are the most common reason for injury-related demise in older grown-ups and a typical cause of attendance at accident and emergency departments. In any case, these patients once in a while underwent rule-coordinated screening and mediations during or following a scene of care. Diminishing damaging falls in a maturing society starts with pre-hospital assessments and proceeds through hazard evaluations and mediations that happen after crisis division care. Even though means for preventing people from needing to access emergency services have been implemented, proof-based systems to decrease the number of falls in elderly adults rely on fall prevention, and advancements incorporate the approval of screening instruments and the consolidation of contemporary innovations, such as PDAs (personal digital assistants), to improve fall location identification rates. This work [78] included measures that speak to various elements (clinical versatility and parity, quality, physiological, postural influence, and the mean and fluctuation of distinction scores among double- and single-task walk conditions) to decide the blend of measures that were the most sensitive for distinguishing fallers from non-fallers. This study aimed to analyze a smartphone fall prevention app to identify product features [79]. Along with that, the scope of revenue generation was also explored using willingness to pay (WTP).

### 3.6. Threshold- and Machine Learning-Based Approach

To develop a reliable and accurate fall detector, it is desirable to have a system that is capable of effectively distinguishing ADL from falls. The authors in [80] developed a paradigm that utilizes the sensors of a smartphone. Advanced signal processing procedures were used to obtain the moving average of scalar values of the three accelerometer components. The adoption of the cross-correlation event polarized approach helped the system to behave robustly. For better classification, two different types of classification algorithms were used, one based on threshold mechanism and the other on principal component analysis (PCA). The performance of the paradigm can be analyzed on two aspects, namely, the classification of a fall and distinguishing a fall from ADL. As compared to the threshold-based approach, the method outperformed on both aspects. However, the performance was moderate for the classification of falls and satisfactory for distinguishing falls from ADL. To improve the performance of the classification of falls, a modified classifier was presented in [80]. In the modified classification approach, the posture information of the user was also gathered after the ADL detection. Using this information, it was easy to discriminate between the multiple classifications of the same event, which was made feasible when using a large dataset for assessment.

In [81], a low-cost and very accurate fall detection algorithm based on machine learning has been proposed. A new method for online feature extraction which employs the fall’s time characteristics efficiently has been proposed. Along with the same, a new design of a system based on machine learning has been proposed which can achieve the numerical/accuracy complexity tradeoff. The lower computing cost of the algorithm helps to combine it with a wearable sensor as well as make the requirement of energy much lower, which increases the wearable device autonomy. The experimental results on a big open dataset show that the accuracy of the proposed algorithm is 99.9% with a computing cost of less than 500 floating-point operations per second. The fall detection systems that utilize the built-in accelerometer sensors of smartphones have been developed to overcome several limitations. One of the major drawbacks of these systems is the enhanced false alarm rate that inhibits their use as a preferred approach. In this work [82], a new technique has been proposed using data mining for monitoring falls. The accelerometer data is mined to discover sequence patterns. These patterns are utilized to formulate a robust system for monitoring falls based on the mobile platform. The proposed solution was tested on a real dataset as well as the MobiFall dataset. The results were compared with existing fall detection algorithms that are smartphone based, and it was found that the method achieved an acceptable false alarm rate. Fall detection was improved using consecutive-frame voting in this work [83]. The process starts with human detection using background subtraction. The subtraction was conducted using a combined approach that involved a mixture of the Gaussian model with an average filter model. The feature extraction section has the task of calculating orientation, aspect ratio, and area ratio from the PCA (principal component analysis) of a human silhouette. In the human centroid section, the moving objects were grouped using human centroid distance. In event classification, event postures are classified. In the end, the voting of majority results is counted from consecutive runs. The results with improved accuracy indicate that the proposed method is better than the prior work that was tested on the Le2i dataset. Most of the techniques are based on a TBA (threshold-based algorithm). However, some researchers have used machine learning-based approaches to predict falls. The hybrid approach of TBA and ML are available in some cases, but each method has its strengths and shortcomings. The work completed in [84] analyzes the TBA- and/or ML-based techniques. The work performed in [85] is capable of identifying the pattern of falls along with the task of detection. This information regarding patterns is further utilized for assistance using machine learning. The proposed method was successful at efficiently differentiating falls from non-falls, thereby increasing accuracy. An automated method for inspection is proposed in this paper [86] to check PPE (personal protective equipment) usage by steeplejacks mounted beside exterior walls for aerial work. The inclusion of the aerial operation scenario-understanding method makes the inspection a tool that can be used to take preventive measures for control. The occlusion mitigation method based on deep learning is used for PPE checking. The method was tested under various conditions. The demonstrations and experimental results proved the reliability and effectiveness of the method for fall prevention and help in adopting safe supervision. The important offering of work [87] is a non-linear model along with threshold-based classification for recognizing abnormal gait patterns with more accuracy. Within the same paper, a dataset with some real parameters was developed to calculate fall prediction. The smartphone sensors of the gyroscope and accelerometer have been used for dataset creation. The presented approach has been implemented and an accuracy of 93.5% has been achieved, which is good compared to other approaches.

### 3.7. Other Approaches

Sannino et al. [88,89] proposed an approach where a tag is placed on the subject’s chest for providing data. The concept of windowing was used to classify windows in fall and non-fall action categories. Consequently, a final window composition was used to determine the global action as a fall or non-fall. The technique was tested and verified on real data comprising fall and non-fall events. The testing results were convincing and justified the effectiveness of their approach. The work presented in [90] elaborates upon the multi-player fall prevention game platform and fall sensing games that were inspired by the exercise program of Otago. The results of the work showed that the game integrates well with senior care centers. Another work, Ref. [91] presented an improvement of Kalman filter-based slip estimation for characterizing slipping distance. The very impressive thing about the algorithm is the detection of accurate slip onset in a fast manner along with the cost-effective and non-intrusive features of the sensor. For the validation and demonstration of the implemented work of a slip detection and estimation model, several experiments have been conducted. The work given in [92] presented a wireless channel data-based fall-sensing system that is real time and transparent. A dynamic template matching (DTM) algorithm has been utilized to build up FallSense. The model has been tested on Wi-Fi devices and an evaluation of the same has been conducted in real environments. The results presented in the work show the outperformance of FallSense compared to other approaches in terms of parameters, such as false alarm rate, complexity, and precision. One of the top reasons for injuries among elderly people is falling. Present solutions suggest wearing fall-alert sensors, but they have been shown to be ineffective in medical research because most of the time elderly people do not wear them. These things became the reason why the new passive sensors that interpret falls using radio frequency (RF) have come into existence. This does not have any implications for elderly people, and it does not encourage them to wear any kind of device. The existing approaches cannot deal with real-world complexities, although major advances have been made in passive monitoring. These approaches perform training and testing on the same people in the same environment, and they cannot extend it to a new environment. Additionally, these approaches cannot differentiate motions from different people, which makes it easy to miss out on a fall in the presence of different motions. To handle these problems, Aryokee, a fall detection system that is RF based [93] and which utilizes a state machine-governed convolutional neural network was proposed. The fall detection system, Aryokee, works with new environments and people who are not seen in the training set. It also separates dissimilar sources of motion to improve robustness. The dataset used was of 140 people performing activities of 40 types in different environments (57 different environments). The results achieved show 92% precision and 94% recall in fall detection. The methods of fall detection based on wearable inertial devices have been explored from 2013 to 2018 [94]. First and foremost, fall definition, fall’s conventional phases, the categories of falls, and the classification of falls have been introduced completely. The research work has been explained in the context of modules, such as the collection of data, pre-processing, feature extraction, and the construction of a model for wearable fall detection system frameworks. The evaluation of the fall detection method’s performance has been performed by inducing the most-used technical criteria. Finally, nine datasets of fall detection have been elaborated upon, and also the predictive performance based on the datasets has been assessed.

The FLIP (flooring for injury prevention) study [95] was a superiority trial conducted over a random 4 years in 150 single rooms at a Canadian LTC (long-term care) site. Residents’ rooms were randomly blocked (1:1) with compliant flooring installation (2.54 cm smart cells) or rigid control flooring (2.54 cm plywood) covered with hospital-grade vinyl in April 2013. The foremost result was a fall injury of a serious manner lasting more than 4 years which needed a visit of the emergency department and a process of treatment or a hospital diagnostic evaluation. The secondary results included minor injuries, or any injuries related to falling, fracture, and falls. Results were confirmed by blinded assessors between 1 September 2013, and 31 August 2017, and examined with treatment as the objective. The problem of fall detection has been studied elaborately for a long time. However, designing accurate embedded algorithms with affordable computing costs is still a challenge because of limited wearable hardware resources.

This work [96] presents a model that is non-stationary, and which is important for such system development. A 3D stochastic trajectory model has been designed to find the mobility patterns of the user. The designed model has a forward fall mechanism. Radio waves will be transmitted to the complete indoor propagation environment, and the fingerprints of the object scattered on the emitted waves will be collected by the receiver. The radio channel has been modeled correspondingly through a process that captures the Doppler effect based on time spent by the occupant at home. The non-stationary channel’s time-frequency behavior has been studied by calculating the power spectral density of the Doppler effect and with spectrogram analysis. The derivation and simulation of instant mean Doppler shift and spread have been performed and the proposed model showed results at 5.9 GHz. The presented results are very effective at developing fall detection models which are reliable, and the model is helpful for studying the effect of several walking/falling patterns. The results are intuitive for emergent reliable fall detection techniques, though the model is functional for studying the impact of diverse patterns on the whole fall detection system performance.

This research [97] outlines a detailed technique based on CNNs (convolution neural networks) for identifying falls using non-invasive thermal vision sensors. It consists of an agile information compilation for labeling images to produce a dataset that describes numerous cases of both multiple and single occupancies. The cases mentioned comprised situations with a fallen inhabitant and standing inhabitants. They also provide information augmentation methods for optimizing the capability of classification learning and the reduction of configuration duration. Third, they define three types of CNN for analyzing the effect of the number of layers and the size of the kernel on the technique’s performance. The obtained results show, in the context of single occupancy, an accuracy of 0.92, and a reduction of 0.10 in accuracy in multiple occupancies. The learning abilities of CNNs have been highlighted as outstanding for use with composite images gained from the inexpensive tools. Do the thus-produced images have more noise along with uncertain and blurred areas? The result shows that a CNN based on three layers executes stable performance, along with fast learning. The planned technique in [98] offered extracts of motion data using a best-fit approximated ellipse and a bounding box around the human body, finding a histogram projection and identifying head position over time, which is useful for producing ten features for fall identification. The above features are fed into a multilayer perceptron neural network to calculate fall categorization. The investigational outputs explain the reliability of the planned method for a high fall detection rate of 99.60% and a low false alarm rate of 2.62% when used with the UR fall detection dataset. Comparisons to state-of-the-art fall detection methods revealed the robustness of the planned method.

The study conducted in [99] focuses on the validation and improvement of existing algorithms for fall detection. The study was conducted in two phases. In the first phase, twenty subjects were recruited of ages 86.25 ± 6.66 years who had experienced high-risk falls. The data concerning their movements were recorded for 59 days in real time using the AIDE-MOI sensor. The existing algorithms were optimized using these data. Then, the evaluation of the optimized algorithm was performed for 66 days. In total, 31 real falls were recorded through the data gathered in both phases. These data were then segmented into one-minute chunks for categorization as “fall” or “non-fall”. A significant improvement was observed in the sensitivity (27.3% to 80.0%) and specificity (99.9957% to 99.9978%) of a threshold-based algorithm. A new method is described in [100] that overcomes several deficiencies of the traditional fall detection methods. The system developed is completely passive and the user is not required to wear any of the devices. The system is developed utilizing the channel state information (CSI) of Wi-Fi along with an accelerometer mounted on the ground to detect floor vibration. The proposed method also overcomes the limitations of existing methods based on the Wi-Fi CSI approach that mandates the presence of only one user in the room. The experimental results show an efficient result of 95% accuracy. A fuzzy logic-based adjustable autonomy (FLAA) model is proposed in [101,102] to handle the autonomy of multi-agent systems that are active in tough surroundings. This model focuses on the management of the autonomy of agents and enables them to make competent autonomous decisions. The autonomy is quantitatively measured and distributed among several agents using fuzzy logic based on their performance.

**Figure 6:**
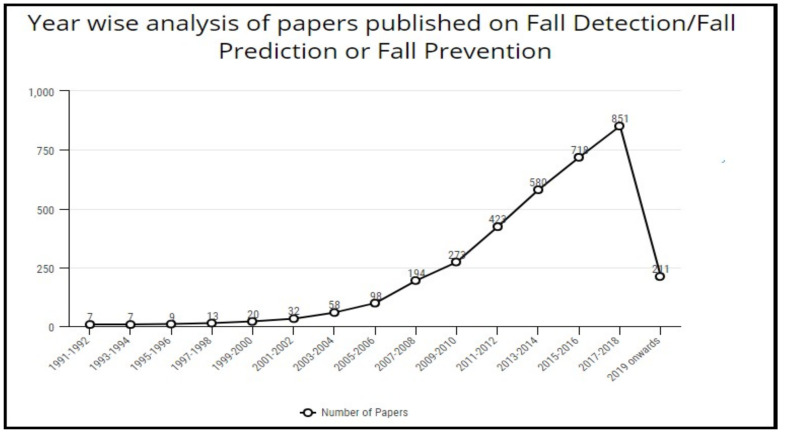
Variation of the number of publications (per publisher).

**Figure 7:**
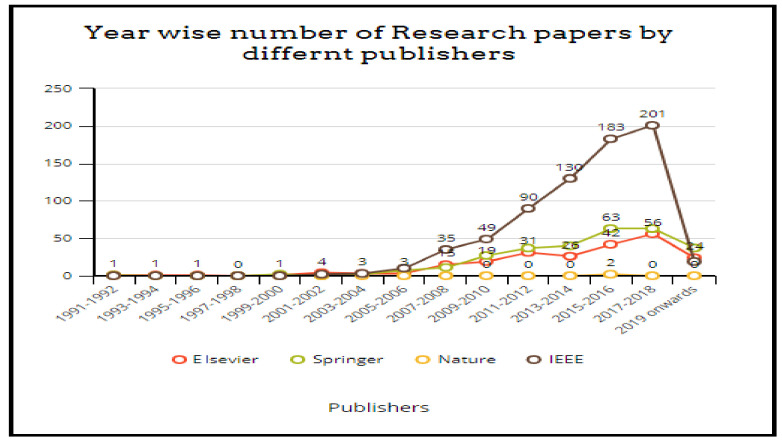
Variation of the number of publications (publisher-wise).

Figure 6 details the variation in the number of publications every two years since 1991. The results for the same are obtained through Google Scholar for the keywords “Fall Prediction” OR “Fall Detection” OR “Fall Prevention”. Similarly, Figure 7 shows the publication details for certain top-level publishers every two years. From the graphs, it is evident that the task of reducing or minimizing the fall risk and its after effects has been motivating more researchers every year. Certain challenges need the focus of active researchers and show the pathways for future research.

**Figure 8:**
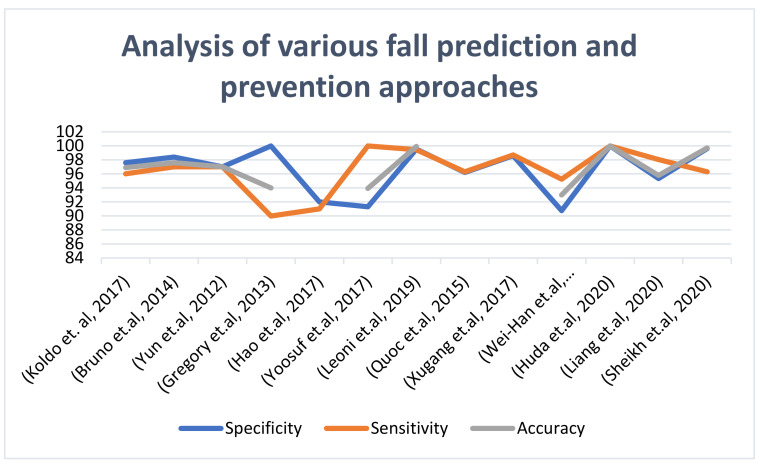
Qualitative analysis of various fall prediction and prevention techniques.

Figure 8 represents [103,104,105] the evaluation of the different approaches developed to detect or prevent falls. The evaluation has been conducted based on the attainment percentage of three parameters: sensitivity, specificity, and accuracy. It can be observed that in some cases, the respective authors succeeded in achieving more than a 98% value for the respective parameters [14,18,106,107,108,109,110,111,112].

## 4. Patents

**Figure 9:**
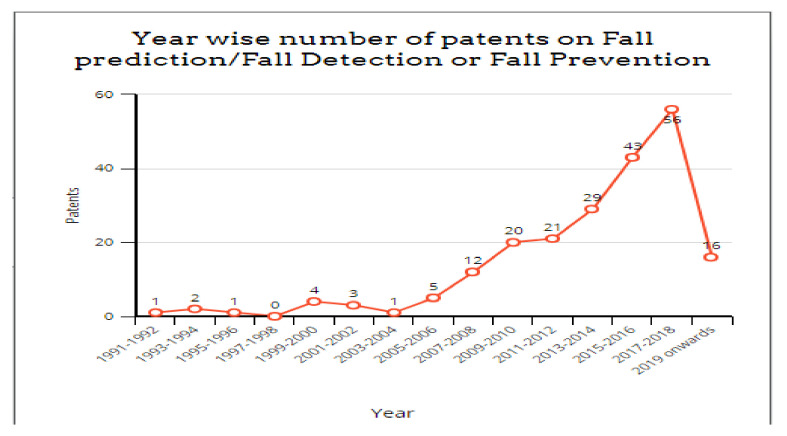
Patents granted on fall prediction or detection.

Researchers have been continuously working for the last three decades to reduce the risk and impact of falls in older people or patients. However, a comparatively fewer number of patents have been filed in this domain. The same is evident in Figure 9. The work conducted in [26] shows the number of patents filed every two years since 1991 to date. Most of the patents are filed in the USA. However, [10] describes the details of 0some of the patents granted in the USA and India. Table 2 gives an insight into some of the patents that have been granted in this domain.

## 5. Projects and Surveys

According to the National Council of Aging, an older adult dies because of a fall every 19 minutes, and every 11 minutes, an older adult is treated in an emergency department for a fall-related injury [101]. Approximately USD 50 billion is spent on treating fall-related injuries in older adults in America. Table 3 describes some projects sanctioned in this domain along with the funding details. Having a birds’ eye view of medical expenditure on falls worldwide is enough to understand the need for projects and research to be carried out in this domain. The OU College of Nursing earns a grant of USD 1 million to continue its fall prevention program. Congress was requested to allocate a budget of USD 10 million for fall prevention programs in just one financial year [112,114,115,116,117,118,119,120,121,122,123].

## 6. Observations and Findings

The systematic study of relevant literature in the field of fall detection and prediction yields a few observations. These findings are the challenges that researchers willing to work in this domain might focus upon.

(i) The majority of the systems developed for detecting or predicting falls in elderly or ambulatory persons are not tested in the real environment. The testing of these systems is primarily performed on the volunteers, who are healthy and young, and usually in the laboratory. The lack of validation against actual users puts doubt on their performance in real life.
(ii) The final acceptance of any system by the actual users is more likely if their opinions are incorporated at the initial stage of development. Unfortunately, the requirements are not gathered by actively involving the elderly peoples initially.
(iii) Most of the projects, patents, and models developed validate their product by measuring certain parameters. There are hardly any cases where user acceptance or satisfaction is taken as the criteria for the effectiveness of the research work conducted.
(iv) A hybrid approach of wearable, as well as ambient devices under reasonable cost would be beneficial to deal with obtrusive factors.
(v) Most of the people who are under consideration are reluctant to press the panic button after a fall. It happens either because of difficulty in activating it or because they do not want to disturb their caregivers.
(vi) Nearly no studies have so far involved the inputs of actual subjects and their relatives and family members. It may be the case that not every time a person falls requires the emergency services. Similar issues can be handled if they are actively involved in the requirement gathering step.
(vii) Usually, the products are designed from a technological perspective, considering things such as power consumption, battery backup, response time, sensors mounting, etc. Medical grounds are surpassed generally by these technical debates.
(viii) In the devices with a push-button, the older people take more time to realize that they are falling rather than younger ones (who are used for testing the device). Consequently, they might not press the button in a timely manner. This is challenge for older people that needs to be addressed.
(ix) The existing systems are hardly in line with the patient confidentiality standards and regulations of the HIPPA.

## 7. Conclusions and Future Scope

Despite continued research over many decades into preventing and predicting falls in elderly people, some factors are still unattended to. The concerns of various governments and the reputed organizations, such as the WHO (World Health Organization), regarding the increasing incidents of falls and their impact are enough to attract researchers to this field. However, some recent research has claimed to achieve the required accuracy in predicting falls, but still they are questionable because of their testing environment. Most of the researchers have not taken into account the perceptions of the actual users regarding what they expect from the product. National governments prefer to give funding for promoting research in this field so that the budget that is spent on after-fall services can be reduced. In the future, the researchers may focus on exploiting some of the principal observations stated in this paper. A hybrid approach of proper education, IoT techniques, and clinical support is expected to achieve real goals.

## Figures and Tables

**Table 1:** Performance of accelerometer-based fall detection devices [4,5,6,7,8,9,10,11,12,13,15,16,17,19,20,21,22,23,24,25,26,27,28,29,30,31,32,33,34,35,36,37,38,39,40,41,42,43,44,45,46,47,48,49,50,51,52].

Title	Author Details	Year	Specificity	Sensitivity
Evaluation of accelerometer-based fall detection algorithms on real-world falls	F. Bagalà et al.	2012	83.3	57
Evaluation of a threshold-based tri-axial accelerometer fall detection algorithm	A.K. Bourke et al.	2007	91.6	93
Comparison of low-complexity fall detection algorithms for body attached accelerometers	M. Kangas et al.	2008	100	98
Accurate, fast fall detection using gyroscopes and accelerometer-derived posture information	Q. Li et al.	2009	92	91
Barometric pressure and triaxial accelerometry-based falls event detection	F. Bianchi et al.	2010	96.5	97.5
Assessment of waist-worn tri-axial accelerometer-based fall-detection algorithms using continuous unsupervised activities	A. Bourke et al.	2010	100	94.6
A wearable pre-impact fall detector using feature selection and support vector machine	S. Shan et al.	2010	100	100
Unsupervised machine-learning method for improving the performance of ambulatory fall-detection systems	M. Yuwono et al.	2012	99.6	98.6
Evaluation of fall detection classification approaches	H. Kerdegari et al.	2012	92	90.15
Patient Fall Detection using Support Vector Machines	C. Doukas et al.	2007	96.7	98.2
A framework for daily activity monitoring and fall detection based on surface electromyography and accelerometer signals	J. Cheng et al.	2013	97.66	95.33

**Table 2:** Details of patents granted [113].

S. No.	Patent ID	Patent Title	Year of Approval	Inventor Name	Country
1	US10037669B2	Fall detection technology and reporting	2018	Mark Andrew Hanson, Jean-Paul Martin, Adam T. Barth, Christopher Silverman	USA
2	US8990041B2	Fall detection	2010	Mark D. Grabiner, Kenton R. Kaufman, Barry K. Gilbert	USA
3	US20160100776A1	Fall detection and fall risk detection systems and methods	2015	Bijan BolooriNajafi, Ashkan Vaziri, Ali-Reza	USA
4	US20180263534	Fall detection device and method for controlling thereof	2018	Han-sung Lee, Jae-geol Cho, Moo-rim Kim, Chang-hyun Kim	USA
5	US20180146737	Shoe system for the detection and monitoring of health, vitals, and fall detection	2018	Joseph Goodrich	USA
6	US20180007257	Automatic detection by a wearable camera	2018	Senem Velipasalar, Mauricio Casares, Akhan Almagambetov	USA
7	2316/CHE/2013	System And Method For Personal Crash/Fall Detection And Notification	2013	Abhishek H Latthe	INDIA

**Table 3:** Details of funded projects for fall detection or prevention.

Project Title	Investigators	Year of Sanction	Organization	Funding Details	Project Description
“Randomized Trial of a Multifactorial Fall Injury Prevention Strategy: A Joint Initiative of PCORI and the National Institute on Aging of the National Institutes of Health” [61]	Shalender Bhasin, Thomas Gill, David B. Reuben	2014	Harvard Medical School; Yale Medical School; UCLA Medical School	Budget: $33,365,602 Source: Patient-Centered Outcomes Research Institute	Behavioral Interventions, Care Coordination, Other Clinical Interventions, Other Health Services Interventions, Technology Interventions, Training and Education Interventions
“Home Safety Adaptations for the Elderly (Home SAFE)” [62]	Unspecified	2010	Fall Prevention Center of Excellence, headquartered at the University of Southern California Leonard Davis School of Gerontology	Budget: Unspecified Source: The Eisner Foundation	Home safety for older people from Fall, fire, etc. and develop and implement related strategies
“Design and Development of fall prediction and protection system for pelvis & femur fractures: Preliminary study” [63]	Dr. Dinesh Kalyanasundara m	2015	Centre for Biomedical Engineering, Indian Institute of Technology (IIT)-Delhi, Hauz Khas, New Delhi- 110 016.	Budget: Rs.26,78,162/- Source: DST INDIA	Unspecified
“WIISEL(Wireless Insole for Independent and Safe Elderly Living)” [124]	Fanny Breuil, Meritxell Garcia Milà	2007	WIISEL, 7th Framework Programme	Budget: $2.9 M Source: European Commission	To prevent falls in older people
“Development of a wireless sensor network based gait assessment system for fall predictionin elderly patients” [125]	Prof. Subrat Kar	2008	Bharti School of Telecommunication Technology and Management, Indian Institute of Technology Delhi, Hauz Khas, New Delhi- 16	Budget: Rs.36,73,200/- Source: DST INDIA	Unspecified

## Data Availability

The data presented in this study are available on request from the corresponding author.
